# *Klf15* Is Critical for the Development and Differentiation of *Drosophila* Nephrocytes

**DOI:** 10.1371/journal.pone.0134620

**Published:** 2015-08-24

**Authors:** Jessica R. Ivy, Maik Drechsler, James H. Catterson, Rolf Bodmer, Karen Ocorr, Achim Paululat, Paul S. Hartley

**Affiliations:** 1 University of Edinburgh / British Heart Foundation Centre for Cardiovascular Science, Queen’s Medical Research Institute, 47 Little France Crescent, Edinburgh, EH16 4TJ, United Kingdom; 2 Department of Zoology & Developmental Biology, University of Osnabrück, Barbarastr. 11, D-49069 Osnabrück, Germany; 3 Neuroscience and Aging Research Center, Sanford-Burnham Medical Research Institute, 10901 North Torrey Pines Road, La Jolla, CA 92037, United States of America; 4 Department of Life and Environmental Science, University of Bournemouth, Talbot Campus, Poole, Dorset BH12 5BB, United Kingdom; University of Valencia, SPAIN

## Abstract

Insect nephrocytes are highly endocytic scavenger cells that represent the only invertebrate model for the study of human kidney podocytes. Despite their importance, nephrocyte development is largely uncharacterised. This work tested whether the insect ortholog of mammalian Kidney Krüppel-Like Factor (Klf15), a transcription factor required for mammalian podocyte differentiation, was required for insect nephrocyte development. It was found that expression of *Drosophila Klf15* (*dKlf15*, previously known as *Bteb2*) was restricted to the only two nephrocyte populations in *Drosophila*, the garland cells and pericardial nephrocytes. Loss of *dKlf15* function led to attrition of both nephrocyte populations and sensitised larvae to the xenotoxin silver nitrate. Although pericardial nephrocytes in *dKlf15* loss of function mutants were specified during embryogenesis, they failed to express the slit diaphragm gene *sticks and stones* and did not form slit diaphragms. Conditional silencing of *dKlf15* in adults led to reduced surface expression of the endocytic receptor Amnionless and loss of *in vivo* scavenger function. Over-expression of *dKlf15* increased nephrocyte numbers and rescued age-dependent decline in nephrocyte function. The data place *dKlf15* upstream of *sns* and *Amnionless* in a nephrocyte-restricted differentiation pathway and suggest *dKlf15* expression is both necessary and sufficient to sustain nephrocyte differentiation. These findings explain the physiological relevance of *dKlf15* in *Drosophila* and imply that the role of *KLF15* in human podocytes is evolutionarily conserved.

## Introduction

Invertebrates, like mammals, have both phagocytic and non-phagocytic scavenger systems for the clearance from circulation of effete cells, foreign material and macromolecules [1–3]. In *Drosophila* the non-phagocytic system is comprised primarily of nephrocytes, highly endocytic cells that exists close to the heart (pericardial nephrocytes) and oesophagus (garland cells). Nephrocytes have long been regarded as analogous to mammalian reticuloendothelial cells [4], and recent studies indicate they express genes conserved in human renal podocytes (e.g. *sticks and stones*, an ortholog of mammalian *Nphs1*, which is critical for slit diaphragm formation) [5, 6] and renal proximal tubule cells (*Amnionless* and *Cubulin*, genes critical for protein reabsorption and endocytosis in invertebrates and mammals)[7, 8]. As such, nephrocytes represent an ancestral blood filtration system whose molecular genetics has been conserved, yet repositioned in different mammalian cell types. The molecular genetics of nephrocyte development and differentiation has not been studied in detail and no transcriptional networks have been identified that control the post-embryonic differentiation of nephrocytes. Identifying such networks in *Drosophila* is important because they may provide important insights into genetic pathways relevant to human physiology and stem cell differentiation.

The Krüppel-like family (KLF) of transcription factors mediate the development, differentiation and function of multiple cell types from invertebrates to mammals [9]. KLF genes encode for proteins with a highly-conserved C-terminal triple tandem repeat zinc finger DNA binding domain and a highly variable N-terminal region. The human ‘kidney KLF’ (KLF15) has a rodent ortholog that regulates cardiac and renal fibrosis, circadian nitrogen metabolism and cardiac potassium channel expression, cardiac hypertrophy, adipogenesis and renal podocyte differentiation [10–15]. An ortholog of *KLF15* exists on the X-chromosome in *Drosophila* but this gene was originally regarded as an ortholog of mammalian *Bteb2/Klf5*, hence it was named *Bteb2*. However, this gene shares greater amino acid sequence similarity with the more recently identified mammalian *Klf15* [16] (see S1 Fig), and we refer to the *Drosophila* gene as *dKlf15* to reflect this. Embryonic and adult gene expression data indicate that *dKlf15* has a highly restricted expression pattern limited to the embryonic garland cells [17] and the adult heart [18], consistent with a role for the gene in nephrocyte development or function (see S2 Fig for expression data).

**Fig 1:**
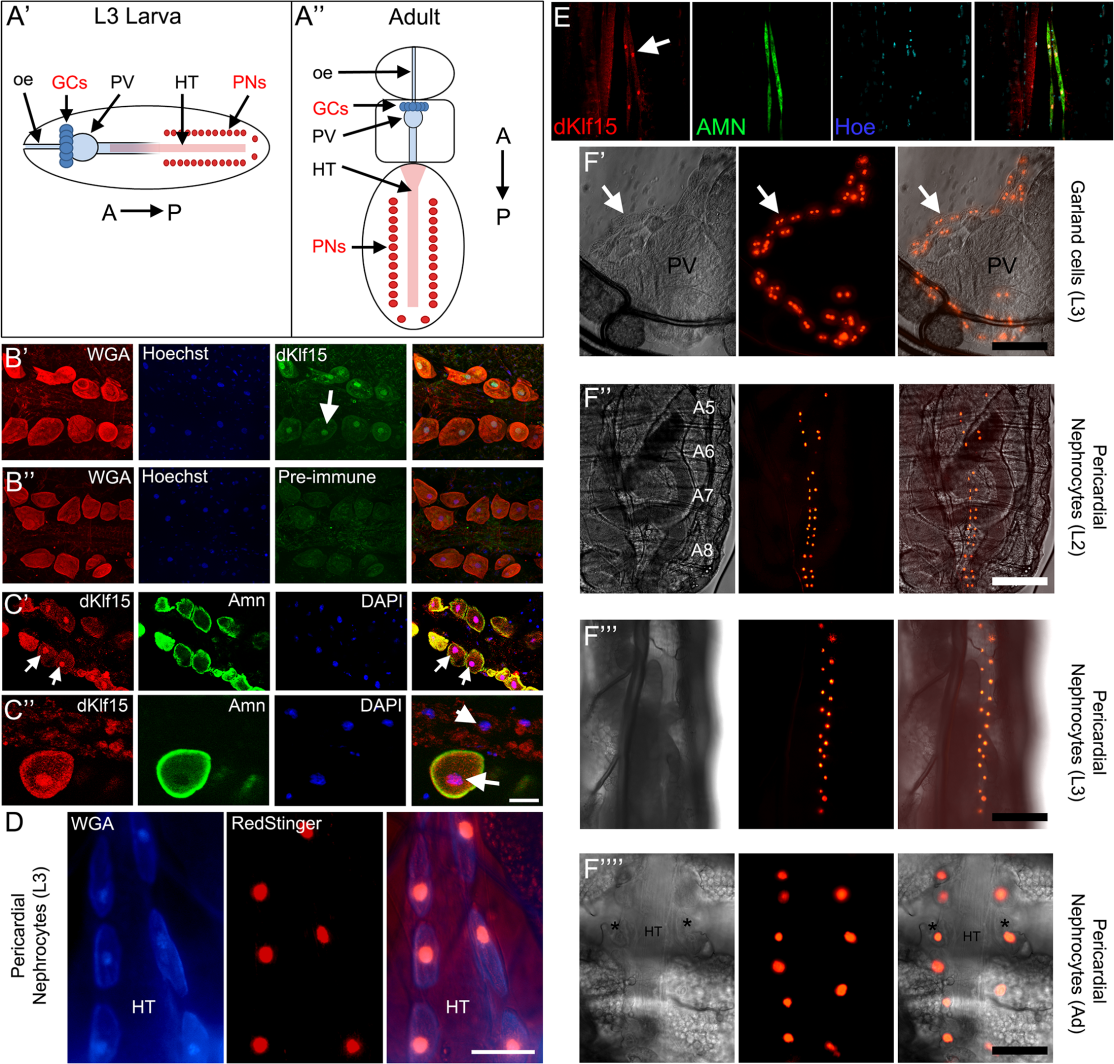
*dKlf15* expression is restricted to nephrocytes. (A’ & A”) Schematics showing location of the two nephrocyte populations in larval and adult *Drosophila*, the garland cells (GCs) and pericardial nephrocytes (PNs). Garland cells are at the interface between the paraventriculus (PV) and oesophagus (oe), whereas pericardial nephrocyte are either side of the heart tube (HT); A>P, anterior-posterior. (B’ & B”) Antisera raised to dKlf15 (but not the pre-immune sera, B”), locate to the nucleus of adult pericardial nephrocytes (arrow). (C) Two magnifications of the adult heart immunostained with anti-dKlf15 (1:10) and anti-Amnionless (1:100) antibodies. Arrows denote pericardial nephrocytes, the arrowhead indicates a cardiomyocyte nucleus. Scale bar = 20 μm. (D) The heart in a dissected L3 larva expressing the RedStinger fluorescent reporter driven by *dKlf15-Gal4* and stained with wheat germ agglutinin which is taken up preferentially by pericardial nephrocytes (blue); HT = heart tube; scale bar = 40 μm. (E) L2 larva stained with anti-Amnionless and anti-dKlf15 showing localisation of dKlf15 to the nucleus of Amnionless-positive pericardial cells (arrow). (F’-F”“) *dKlf15-Gal4* driven *RedStinger* expression in living larvae and a dissected adult. Fluorescence is seen in binucleate garland cells next to the paraventriculus (PV) in L3 larvae (arrows). Fluorescence was also detected in pericardial nephrocytes (asterisks) either side of the heart tube (HT) at L2, L3 and adult (Ad) stages. Scale bars = 50 μm.

In this work we use loss and gain of function studies to test the hypothesis that *dKlf15* may play a cell specific role in nephrocyte differentiation. Loss of function studies identified *dKlf15* as critical for the development of *Drosophila’s* only two populations of nephrocytes, the garland cells and the pericardial nephrocytes (see anatomical schematics in Fig 1A). The expression of the slit diaphragm gene *sticks and stones* as well as the endocytic receptor *Amnionless* were both dependent upon *dKlf15* expression. Focussing attention on pericardial nephrocytes revealed that these cells were specified normally during embryogenesis but then died during larval development. Using a conditional silencing strategy it was established that adult nephrocyte function depended upon sustained *dKlf15* gene expression. Over-expression of *dKlf15* increased nephrocyte numbers and prevented their age-dependent dysfunction. These findings define the physiological relevance of *dKlf15* in *Drosophila* and begin to annotate a genetic pathway driving and sustaining nephrocyte differentiation. The findings also suggest that the role of *Klf15* in mammalian kidney podocytes is evolutionarily conserved and that *Drosophila* may be used to identify novel pathways relevant to the regulation and role of *KLF15* in humans.

## Results

### *dKlf15* is a nephrocyte-restricted transcription factor

High throughput expression data indicated that *dKlf15* expression is limited to embryonic garland cells as well as the adult heart [17, 18]. To verify this, antisera was raised to a peptide corresponding to a region in the N-terminal of *dKlf15* protein and used to stain the adult heart. Antibodies to dKlf15 (but not pre-immune sera) localised to the nucleus of pericardial nephrocytes that co-expressed the nephrocyte marker Amnionless in adults (Fig 1B and 1C) and L2 larvae (Fig 1E). The antibody did not stain the cardiomyocytes (arrowhead Fig 1C”); nor the fat body, ovary or oenocytes (see S3 Fig). A fluorescent reporter protein (RedStinger; dsRed tagged with a nuclear-localisation signal) driven by a 2131bp region of the putative *dKlf15* enhancer was examined in larval and adult flies. Expression of the reporter was limited to pericardial cells in larvae (seen in dissected preparations as large cells either side of the heart tube that preferentially sequester wheat-germ agglutinin (Fig 1D). No expression of the reporter was seen in L1 pericardial nephrocytes, however expression of the reporter was seen in pericardial cells at the L2 stage (see S4 Fig), concomitant with the differentiation of pericardial cells after the L1 stage (see below) and immune-detection of dKlf15 in Amnionless positive pericardial cells (Fig 1E). The reporter was also expressed at L3 and adult stages, as well as in garland cells of L3 larvae (Fig 1F). Expression was restricted to the pericardial nephrocytes at abdominal sections A5-A8. No other cells were seen to express the reporter in adult or larval stages. These findings corroborate the high throughput data and are consistent with *dKlf15* expression being restricted to garland cells and pericardial nephrocytes, the only populations of nephrocytes in *Drosophila*.

### *dKlf15* is critical for nephrocyte differentiation

**Fig 2:**
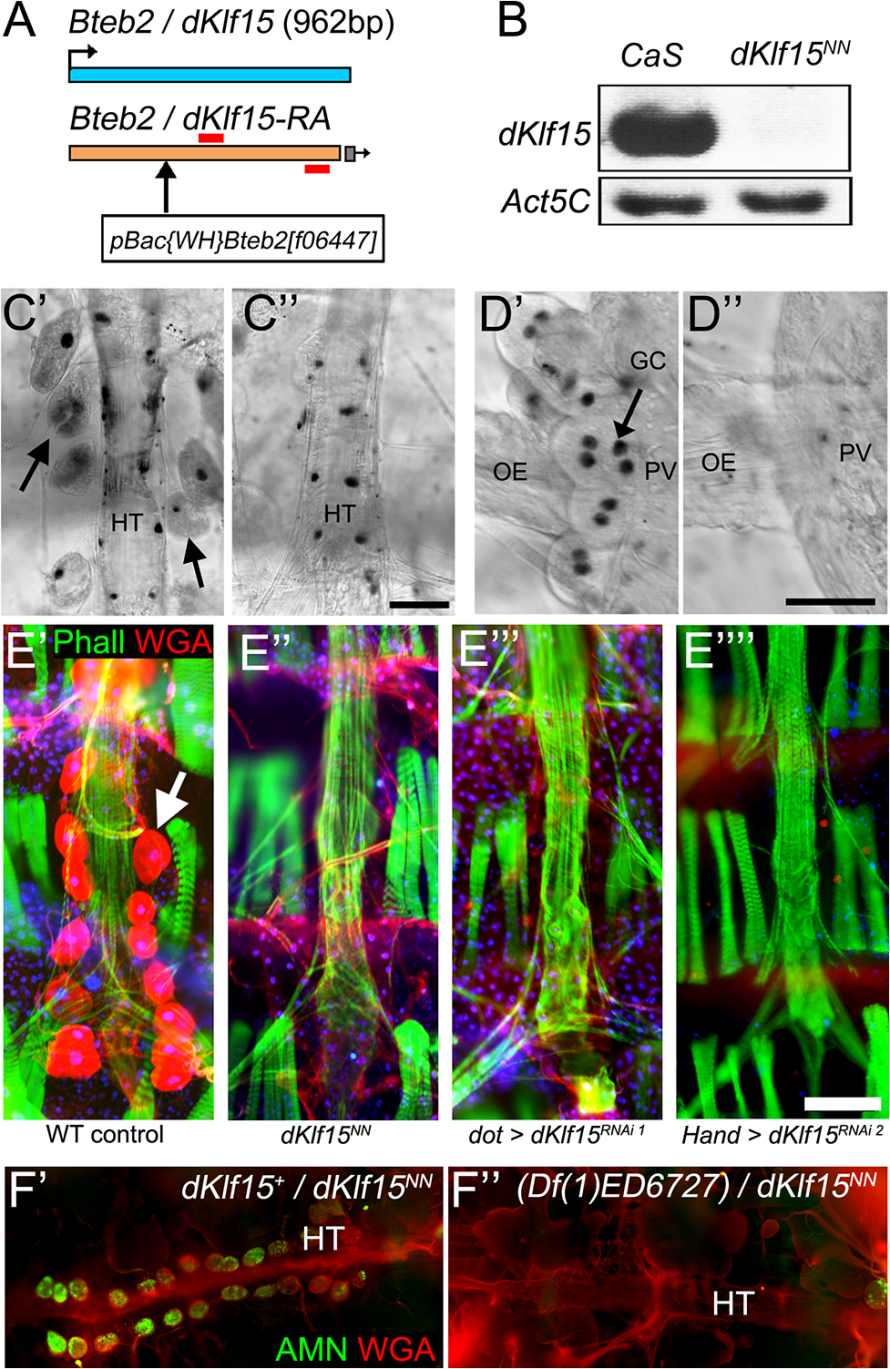
*dKlf15* is critical for nephrocyte development. (A) Schematic of the *dKlf15* gene. An allele of *dKlf15*, originally called *Bteb2* ^*f06447*^, and now renamed *dKlf15* ^*NN*^, contains a piggyBac insertion (white box) within the coding region. The red bars define the primer sequence used to detect *dKlf15* expression. (B) *dKlf15* expression in adult heart from control (*CaS*) and mutant (*dKlf15* ^*NN*^) flies. (C) Phase contrast micrographs of the heart from wild type (C’) and *dKlf15* ^*NN*^ mutant females (C”) overlaid with fluorescence images of a Hoechst-stained adult heart; nuclei are re-coloured black against a white background. Arrows indicate pericardial nephrocytes, HT = heart tube; scale bar = 50 μm. (D) Micrographs of Hoechst-stained larval oesophagus (OE) at the point it meets the proventriculus (PV) in wild type and *dKlf15* ^*NN*^ mutants (D’ % D”, respectively); GC = garland cells (arrow); scale bar = 25 μm. (E) Adult heart from wild type (E’), *dKlf15* ^*NN*^ mutants (E”) or flies where *dKlf15* had been silenced using either *dot-Gal4* or *Hand-Gal4* (E”‘ & E”“, respectively), stained with wheat-germ agglutinin (red) and phalloidin^FITC^ (green); arrow indicates pericardial nephrocytes; scale bar = 100 μm. (F) Adult heart stained with antibodies to Amnionless (green) and phalloidin (red); hearts were from female flies heterozygous for the mutant *dKlf15* ^*NN*^ allele and a wild type copy of the allele (F’) or heterozygous for the *dKlf15* ^*NN*^ mutant allele and a deletion spanning the *dKlf15* locus (F”; (*Df(1)ED6727*)); HT = heart tube.

To establish if *dKlf15* was relevant to nephrocyte biology a line of flies was sourced which had a large insertion within the *dKlf15* locus (Fig 2A). These flies were homozygous viable despite having no detectable *dKlf15* expression (Fig 2B). Analysis of all *dKlf15* ^*NN*^ mutants was performed on offspring from these homozygous mutant parents. Upon dissection of larvae and adults it was evident that binucleate garland cells and pericardial nephrocytes were missing (Fig 2C & 2D). This allele was therefore referred to as *dKlf15* ^NN^, to denote ‘No Nephrocytes’. Silencing *dKlf15* with *dorothy-Gal4* [19] or *Hand-Gal4* [20], led to the absence of nephrocytes in adults (Fig 2E). Female *dKlf15* ^+/NN^ heterozygous flies had a normal number of nephrocytes (Fig 2F), however females transheterozygous for the *dKlf15* ^*NN*^ mutation and a chromosomal deficiency spanning the *dKlf15* locus (*Df(1)ED6727*; i.e. they had no wild type copy of *dKlf15*) had no nephrocytes (Fig 2F).

**Fig 3:**
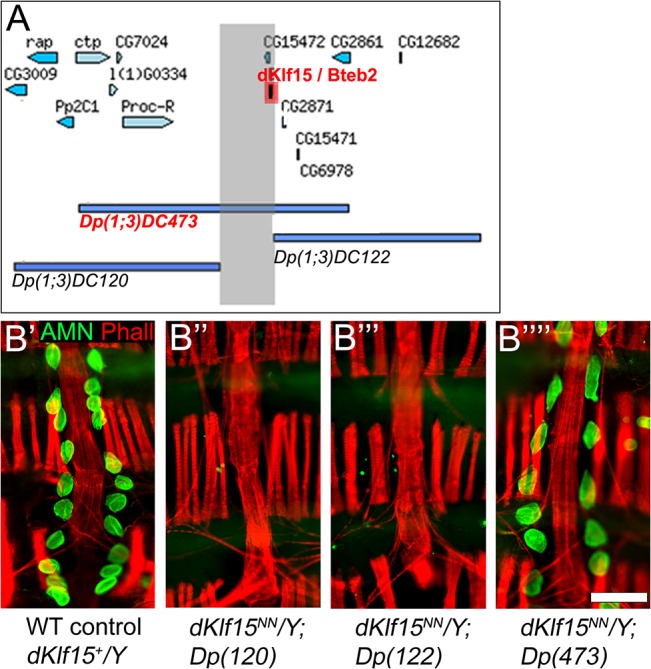
Rescue of the *dKlf15* ^*NN*^ mutation. (A) Schematic showing genomic duplications. (B) Adult hearts stained with anti-Amnionless antibodies (green) and phalloidin (red). Male flies were hemizygous for the mutant *dKlf15* allele (B’); or hemizygous for the mutant allele and a genomic duplication on the third chromosome corresponding to either side of the *dKlf15* locus (*Dp(120)* and *Dp(122*); B” and B”‘, respectively) or covering the *dKlf15* locus (*Dp(473)*; B”“); scale bar = 100 μm.

Adult male flies hemizygous for the *dKlf15* ^NN^ mutation (*dKlf15* is on the X-chromosome) had no nephrocytes (Fig 3A & 3B) and this was rescued by a genomic duplication of the wild type *dKlf15* locus but not duplications spanning regions either side of the *dKlf15* locus (Fig 3A & 3B). These findings established that *dKlf15* is critical for nephrocyte development in *Drosophila*.

### Sensitivity to silver nitrate and lifespan of *dKlf15* ^*NN*^ mutants

**Fig 4:**
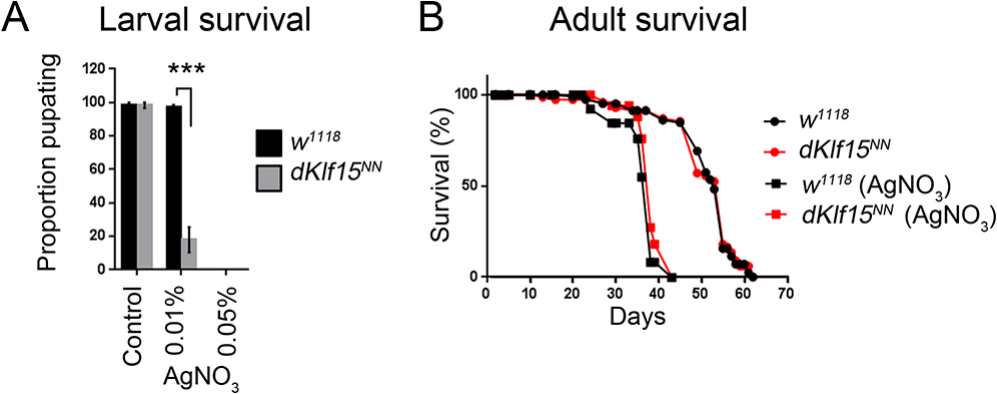
Survival of *dKlf15* mutants. (A) Survival of larvae on yeast containing different amounts of silver nitrate (AgNO3); ***P<0.001; *n* = 3 independent trials starting with 20 eggs per trial. (B) Survival of adult males on control (circles) or AgNO_3_ diet (squares); *n* = 85–120 flies per genotype (housed as 15 flies per vial). Genotype had no effect on lifespan or survival on diet containing silver nitrate (P>0.05). Silver nitrate was toxic to both genotypes (P<0.001, relative to flies on the control diet).

It is known that nephrocytes scavenge ingested silver nitrate and that disruption of nephrocyte function increases mortality of larvae fed silver nitrate [5, 21]. It was therefore hypothesised that loss of *dKlf15* expression may reduce the survivorship of larvae fed silver nitrate. When larvae were fed 0.01% silver nitrate, there was increased mortality of *dKlf15* ^*NN*^ mutants relative to wild-type controls (P<0.01; Fig 4A). There was no difference between genotypes when provided with a normal diet and larvae of both genotypes died when provided with a higher dose (0.05%) of silver nitrate (Fig 4A). Strikingly, the lifespan of adult control (*w* ^*1118*^) and *dKlf15* ^*NN*^ mutant flies was identical (Fig 4B), even when provided with silver nitrate, despite this xenotoxin shortening the lifespan of both genotypes. These findings indicate that *dKlf15* mitigates the toxicity of silver nitrate in larvae but not adult flies and that nephrocytes are dispensable for normal survival under control conditions.

### *dKlf15* is required for the differentiation of post-embryonic pericardial nephrocytes

**Fig 5:**
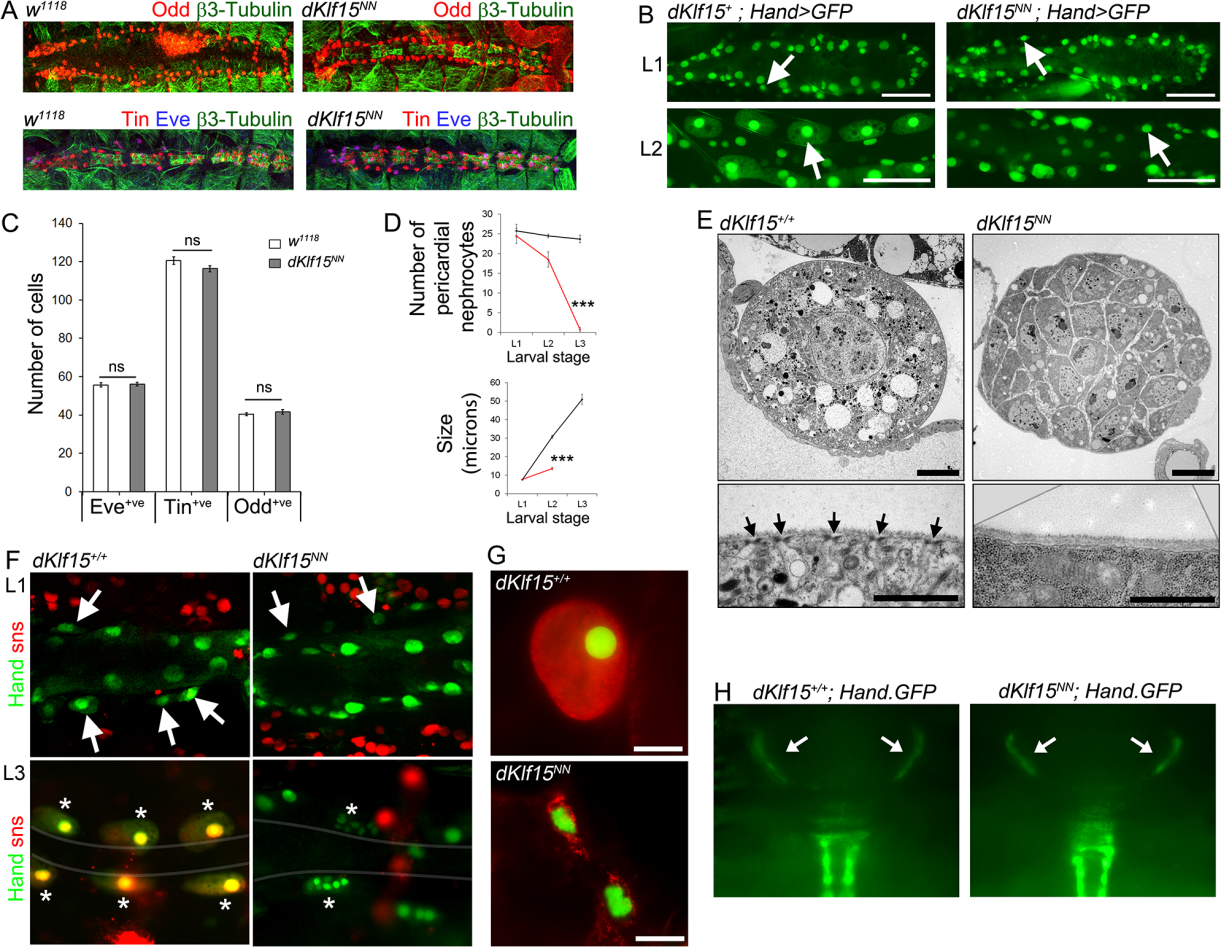
*dKlf15* regulates the post-embryonic differentiation of pericardial nephrocytes. (A) Stage 16 embryos stained with antibodies to Odd-skipped (Odd) or Even-skipped (Eve) and Tinman (Tin) and β3 tubulin. (B) Wild type (*dKlf15* ^*+*^) and *dKlf15* ^*NN*^ on a *Hand-GFP* background to mark cardiomyocytes and pericardial nephrocytes (arrows). (C) Number of Even-skipped, Odd-skipped and Tinman positive cells in stage 16 embryos; *n* = 8–12 embryos per genotype. (D) Number and size of nephrocytes in larvae at different stages. ***P<0.001; *n* = 8–14 larvae per genotype; (note, nephrocyte were too infrequent to quantify at L3 stage). (E) Ultrastructure of nephrocytes from L3 stage wild type (*dKlf15* ^*+/+*^) and mutant *dKlf15* ^*NN*^ larvae. Arrows indicate slit diaphragms. Scale bars = 5 μm (upper panels); = 1 μm (lower panels). (F) *Hand-GFP* wild type and *dKlf15* ^*NN*^ lines expressing a *sticks and stones* reporter (red). Nephrocytes in L1 (arrows); nephrocytes in L3 (asterisks); grey line defines the heart. (G) Nuclear morphology of *Hand-GFP*-positive cells co-stained with wheat germ agglutinin (red), scale bar = 25 μm. (H) Micrographs show the Hand-GFP fluorescence signal of the ‘wing hearts’ (arrows) seen through the cuticle of the scutellum of pupa.

To establish when nephrocyte development was affected by the loss of *dKlf15*, the embryonic heart was stained with antibodies to Tinman, Even-skipped and Odd-skipped, three proteins expressed by cells of the cardiac lineage destined to become pericardial nephrocytes [22, 23]. The number and anatomical location of pericardial nephrocytes in *dKlf15* ^*NN*^ mutants at embryonic stage 16 were normal (Fig 5A and 5C). This suggested that the heart was specified normally in mutants, that there is normally no maternal *dKlf15* contribution and that loss of *dKlf15* function only becomes manifest at a post-embryonic stage of pericardial nephrocyte development. For ease of identification during larval development, control (*w* ^*1118*^) and *dKlf15* ^*NN*^ mutants were crossed into a *Hand-GFP* background to mark cardiomyocytes and pericardial nephrocytes with green fluorescent protein [24]. At the L1 stage the phenotype of the heart in *dKlf15* ^*NN*^ mutants was indistinguishable from that in controls. After L1, the pericardial nephrocytes in controls grew considerably in size, whereas this failed to occur in *dKlf15* ^*NN*^ mutants and by the L3 stage, very few pericardial nephrocytes remained in the mutant (Fig 5B and 5D). dKlf15 protein was detected in wild type L3 pericardial nephrocytes but not *dKlf15* ^*NN*^ mutants (S5 Fig), confirming that the mutant phenotype was due to loss of dKlf15 function.

The filtration slit is a key functional structure in nephrocytes and disruption of the genes which form it leads to nephrocyte death [5]. Transmission electron microscopy was therefore used to examine the ultrastructure of control and *dKlf15* ^*NN*^ mutant nephrocytes. Whereas filtration slits could be readily identified at the surface of pericardial nephrocytes in controls, no such structures were seen in *dKlf15* ^*NN*^ mutants (Fig 5E). Although pericardial nephrocytes were scarce in L3 larvae, those remaining often had a distinctive ‘poly-cellular’ phenotype. The absence of filtration slits prompted an examination of *sticks and stones* expression, a gene required for slit formation [5, 6]. *dKlf15* ^*NN*^ mutant and control *Hand-GFP* lines were therefore crossed with flies expressing fluorescently tagged *sticks and stones* (*sns*). In controls there was no *sns* signal in L1 pericardial cells but strong expression in L2s (S6 Fig) and L3s (Fig 5F). This signal was only seen in the pericardial cells around regions A5-A8, the PNs destined to become the adult pericardial nephrocytes. In stark contrast, no *sns* expression was ever seen in any pericardial cells in the *dKlf15* ^*NN*^ mutants, however *sns* expression was still seen in non-heart cells (S6 Fig), indicating that *dKlf15* ^*NN*^ mutants specifically lacked *sns* expression in the pericardial nephrocytes. The ‘poly-cellular’ phenotype of residual pericardial nephrocytes was also evident in these experiments (asterisks in Fig 5F). Some *Hand-GFP* positive cells in the mutants at the L3 stage exhibited morphology consistent with cell death (compare upper and lower panels in Fig 5G), however by this stage there were very few nephrocytes remaining. In contrast, the pericardial cells located along the aorta and which form the scutellar pulsatile organs (‘wing hearts’ [25]), appeared unaffected by the *dKlf15* ^*NN*^ mutation (Fig 5H). These findings suggest that *dKlf15* regulates the post-embryonic maturation of a subset of pericardial cells destined to become the adult pericardial nephrocytes and that loss of *dKlf15* function leads to the absence of filtration slits, a situation leading to loss of nephrocyte viability. Hence, the lack of pericardial nephrocytes in adult *dKlf15* ^*NN*^ mutants is due to nephrocyte attrition during late larval development.

### Adult nephrocytes’ endocytic scavenger function relies on sustained *dKlf15* expression

**Fig 6:**
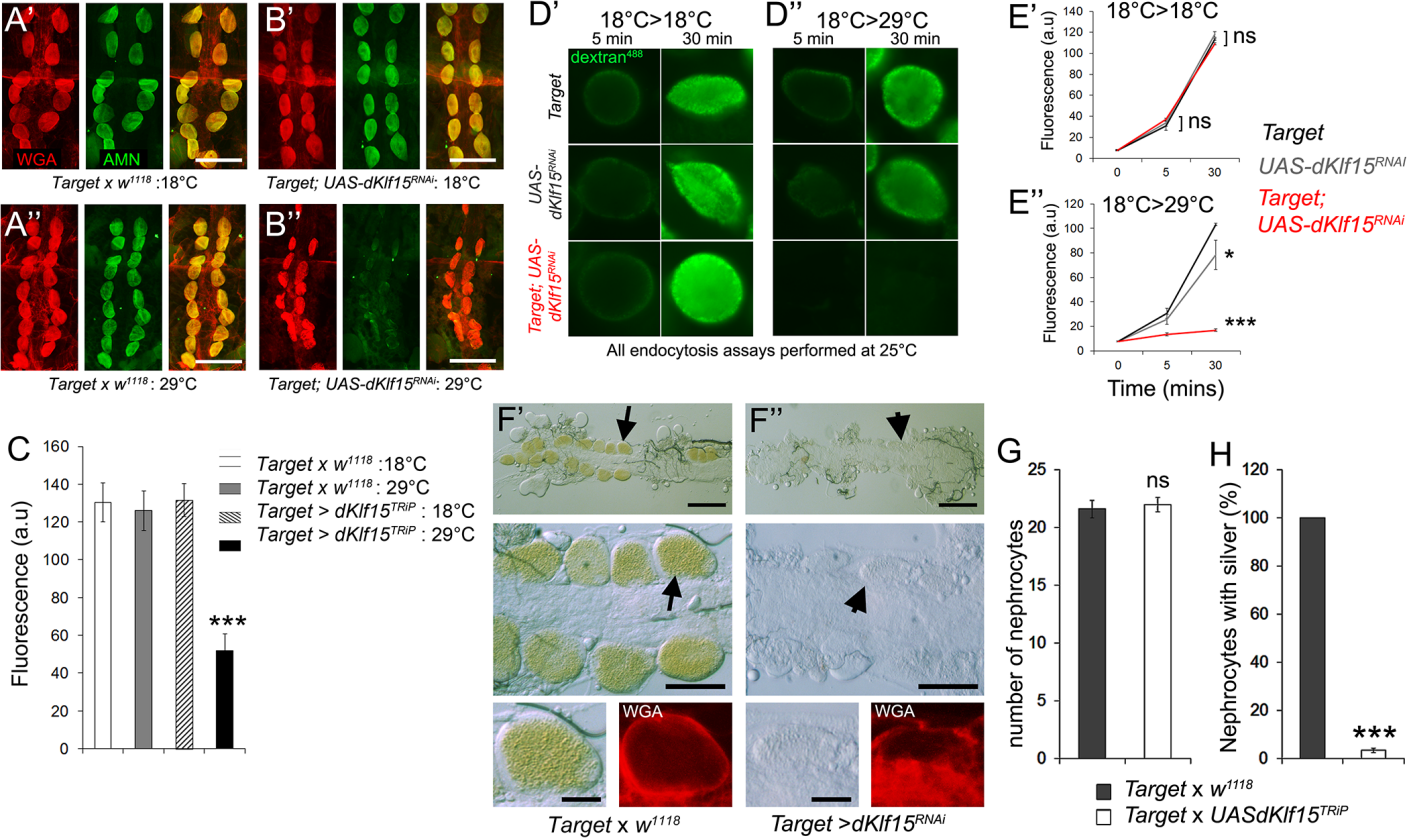
Adult pericardial nephrocyte function is dependent on *dKlf15*. (**A & B**) *dKlf15* was conditionally silenced in adult nephrocytes using Hand-Target (*Target*) to drive *UAS*-*dKlf15* ^*RNAi*^. Flies were reared at 18°C until eclosion then either maintained at 18°C (**A’, B’;** no knock-down) or placed at 29°C (**A”**, **B”**; RNAi permitted) for 4 days. Adult hearts were then stained with wheat germ agglutinin (red) and antibodies to the nephrocyte marker Amnionless (green). Scale bars = 100 μm. (**C**) Quantification of Amnionless fluorescence signal. ***P<0.001; *n* = 8 hearts per genotype. (**D**) Pericardial nephrocytes in semi-intact heart preparations after incubation with fluorescently tagged 10 kDa dextran (green) for 5 or 30 minutes. Nephrocytes of all genotypes associated with dextran when adults were kept at the non-permissive temperature of 18°C (**D’**), whereas at 29°C, the permissive temperature for silencing, the *dKlf15* RNAi line could no longer associate with dextran (**D”**). (**E**) Quantification of fluorescence after incubation for 0, 5 or 30 minutes with fluorescently-tagged. *P<0.05, ***P<0.001compared to control (*Target* genotype), *ns* = not significantly different from control; *n* = 12–16 nephrocytes from 4 individual flies per genotype, per time-point. (**F**) Control (*Target* flies outcrossed to *w* ^*1118*^, (F’)) and *Target* > *dKlf15* ^*RNAi*^ (F”) flies reared at 18°C then transferred to 29°C for 4 days and fed silver nitrate for one week. Upper and middle panels show the pericardial nephrocytes in control flies containing silver (brown pigment, arrows) but not when *dKlf15* had been silenced (arrowheads). Scale bar = 100μm and 50μm. Lower panels show that pericardial nephrocytes of both genotypes could still be identified with wheat-germ agglutinin (red). Scale bar = 20μm. (**G &H**) Quantification of nephrocytes and the percentage of nephrocytes containing silver nitrate. *ns* = not significantly different from the control, ***P<0.001; *n* = 8 flies per genotype.

To establish if *dKlf15* expression mediated nephrocyte function in adult flies, *dKlf15* was silenced in adults using the TARGET system driven by *Hand*-*Gal4*. This system, which exploits a temperature sensitive repressor of Gal4, allows for the temporal and tissue dependent switch-on and off of genes using the bipartite Gal4-UAS system [26]. Flies were raised at the non-permissive temperature (18°C, no RNAi expression) and then either maintained as adults at this temperature or placed at the permissive temperature (29°C) to allow RNAi expression. When *dKlf15* was silenced in adults, immuno-detectable Amnionless was significantly reduced on the surface of the nephrocytes (Fig 6A–6C). Amnionless is known to mediate nephrocyte endocytosis [7] and it was predicted that this would impact on nephrocyte function. Accordingly it was found that the nephrocytes’ endocytic function as tested in dissected preparations of the adult heart (Fig 6D and 6E and also S7 Fig) and ability to scavenge silver nitrate *in vivo* (Fig 6F–6H) were significantly reduced by *dKlf15* knock-down. Hence, the differentiated state and functionality of adult nephrocytes is dependent upon sustained *dKlf15* expression.

### Over-expression of *dKlf15* increases nephrocyte numbers and rescues age-related decline in nephrocyte function

**Fig 7:**
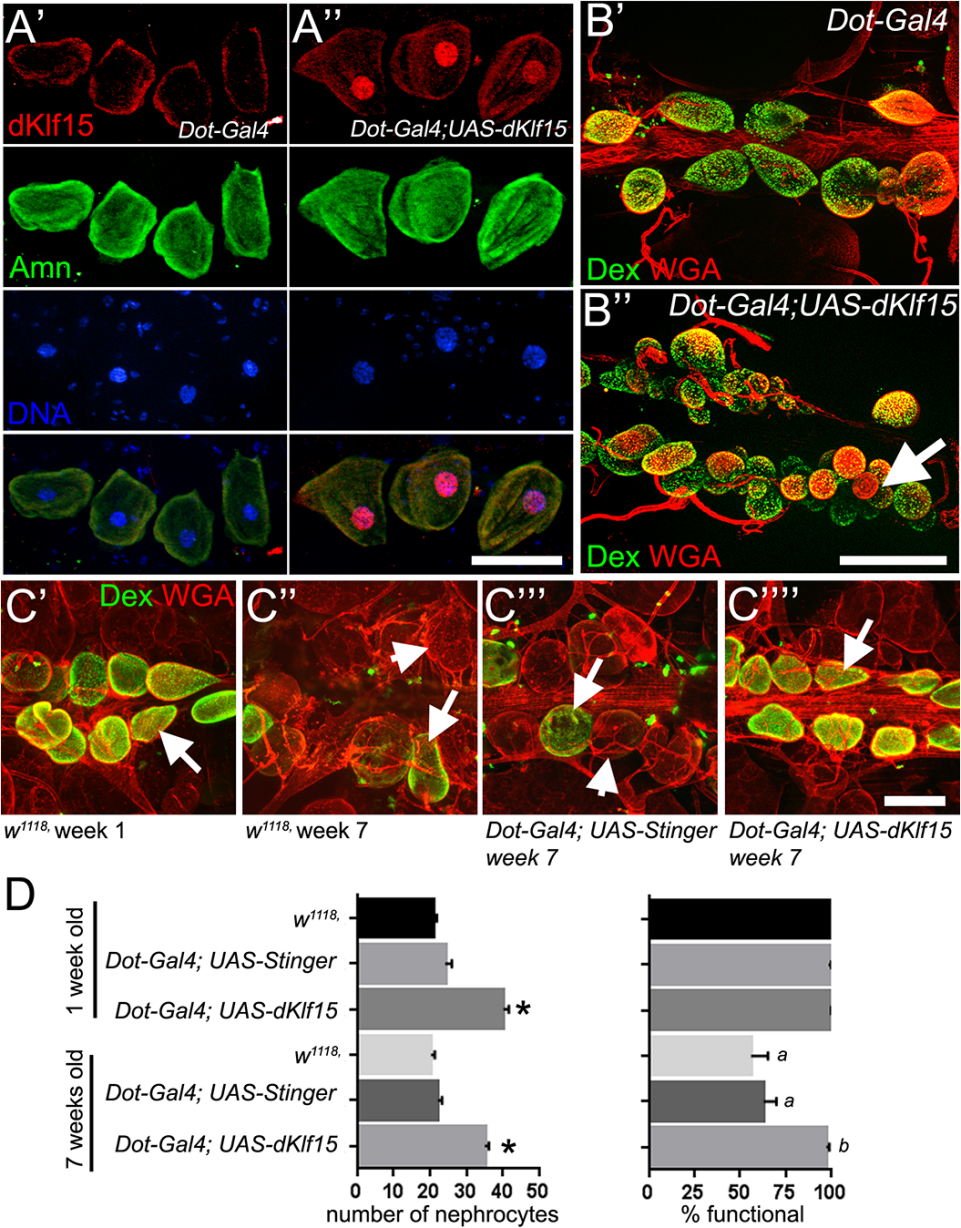
*dKlf15* overexpression increases the number of functional nephrocytes in adults and prevents age-dependent nephrocyte dysfunction. (A) Pericardial nephrocytes from control (progeny of *Dot-Gal4* outcrossed to *w* ^*1118*^; A’) and *dKlf15* over-expressing flies (*Dot-Gal4; UAS-dKlf15*; A”) stained with anti-dKlf15 (1:100) and anti-Amnionless antibodies (1:100). Scale bar = 50 μm. (B’) Pericardial nephrocytes from control (progeny of *Dot-Gal4* outcrossed to *w* ^*1118*^; B’) and *dKlf15* over-expressing flies (*Dot-Gal4; UAS-dKlf15*; B”) incubated with 10 kDa dextran and wheat germ agglutinin (to label cell surfaces). Arrow indicates a group of smaller nephrocytes. Scale bar = 80 μm. (C’-C”“) Nephrocytes from one and seven-week-old flies, incubated with 10 kDa dextran (green) and wheat germ agglutinin (red). Arrows indicate nephrocytes that have accumulated dextran; arrowheads identify nephrocytes that have not accumulated dextran. (D) Quantification of nephrocyte numbers and the proportion which were functional as determined by their ability to bind dextran. *P<0.001 compared to wild type and control; ^*a*^ P<0.001 compared to one-week-old wild type and control; ^*b*^ P<0.001 compared to seven-week-old wild type and control; *n* = 8–12 flies per genotype / per time-point.

During these functional studies it became apparent that nephrocyte function declined as flies aged. Given that *dKlf15* was required for adult nephrocyte function it was hypothesised that *dKlf15* over-expression might rescue the age-dependent nephrocyte dysfunction. Over-expression of *dKlf15* driven by *Dorothy-Gal4* led to an increase in nephrocyte numbers due to the accumulation of small nephrocytes close to the conical chamber (Fig 7). Over-expression of *dKlf15*, despite leading to the production of the population of smaller nephrocyte, did not affect the expression of phenotypic markers (Amnionless) nor the function of the nephrocytes in young (1-week-old) flies (Fig 7A and 7B). All nephrocytes in all genotypes were capable of binding dextran when flies were one week of age (Fig 7C), however there was a significant reduction in the proportion binding dextran at seven weeks (reduced to 56% and 62% of nephrocytes in wild type and control flies, respectively P<0.001; Fig 7D). In stark contrast to nephrocytes in wild type and control flies, there was no such reduction in the proportion of nephrocytes binding dextran in 7-week-old flies over-expressing *dKlf15* (Fig 7D).

## Discussion

Multicellular organisms contain circulating fluids which must be filtered to remove toxins, effete cells and endogenous macromolecules. Whether this is mammalian blood or invertebrate hemolymph, elaborate filtration systems have evolved which maintain organ homeostasis. In insects the principal filtration cell is the nephrocyte. Nephrocytes have long been regarded as functionally analogous to highly endocytic scavenger cells within the mammalian reticuloendothelial system [4, 27, 28]. Recent reports support this, indicating that there is significant genetic overlap between insect nephrocytes and podocytes, a highly specialised cell-type crucial for glomerular filtration in the human kidney [5, 6, 29, 30]. Nephrocytes also express *Amnionless* and *Cubilin*, which form a receptor complex required for nephrocyte filtration function and which is expressed by highly endocytic mammalian cell types, including kidney proximal tubule cells [7]. Hence, the similarities between invertebrate and vertebrate filtration systems appear to share a common genetic basis. The current work reinforces this view by demonstrating that the insect ortholog of Kidney Krüppel-like factor (*Klf15*), a gene required for human renal podocyte differentiation, acts upstream of *Amnionless* and the slit diaphragm gene *sticks and stones*, to drive and maintain the differentiation of *Drosophila* nephrocytes.

In contrast to the restricted pattern of *dKlf15* expression in *Drosophila*, mammalian *Klf15* is expressed in a variety of tissues, suggesting diversification of the ortholog’s role during evolution. Although transcriptional activity of *dKlf15* has been demonstrated in two independent, *in vitro* studies [31, 32] the specific genomic targets in insect nephrocytes remain undefined. Human KLF15 is known to directly activate *NPHS1* and *NPHS2* [13], which encode Nephrin and Podocin, proteins critical for podocyte slit diaphragm formation. Given that *dKlf15* is restricted to nephrocytes, the only insect cell type known to form filtration slits, it is plausible the role of mammalian *Klf15* in podocyte biology has been evolutionarily conserved and that dKlf15 may directly activate *sns*, the *Drosophila* ortholog of human *NPHS1*. This hypothesis is consistent with the observed loss of *sns* expression and lack of slit diaphragms in the pericardial nephrocytes of *dKlf15* ^*NN*^ mutants. Garland cells are known to develop as mononuclear cells which then fuse to form binucleate, mature nephrocytes. This fusion process is dependent on *sns* and modulation of fusion can lead to multi-nucleate garland cells [6, 30]. Overexpression of *neph* genes as well as hyperactivation of Src64B, a kinase that regulates the slit-diaphragm protein encoded by *Dumbfounded*, can lead to multinucleate garland cells through agglutination, a phenotype reminiscent of the poly-nucleate morphology seen in *dKlf15* ^*NN*^ mutants [29, 30]. How the loss of *dKlf15* function leads to a phenotype similar to that seen in gain-of-function experiments is unclear.

Expression of a fluorescent reporter gene (*RedStinger*) driven by the presumed *cis* acting enhancer element of *dKlf15* was limited to garland cells and pericardial nephrocytes. This corroborated high throughput datasets and anti-dKlf15 immuno-staining results, suggesting the enhancer element used to drive *RedStinger* provided faithful representation of the *dKlf15* expression pattern. Attempts to perform *in situs* on larval tissue were unsuccessful, reflecting the possibly that *dKlf15* expression is too low to be detected, so it is not known with certainty when *dKlf15* expression is switched-on in pericardial nephrocytes. However, the timing of nephrocyte attrition and *RedStinger* reporter gene expression, from the L2 stage onwards, would strongly suggest that *dKlf15* expression is initiated in pericardial nephrocytes soon after the L1 stage. No attempt to study the timing of *dKlf15* expression in embryonic garland cells was made but high throughput data indicates *dKlf15* is expressed in garland cells after embryonic stage 10 [17]. Given that *dKlf15* was required for the differentiation and sustained function of pericardial nephrocytes it will be interesting to examine how *dKlf15* affects the development and function of the garland cells.

Garland cells and pericardial nephrocytes develop in different anatomical locations, at different times, suggesting that at least two independent signalling pathways initiate *dKlf15* expression during development. In addition, one of these pathways or possibly a third pathway regulates the sustained expression of *dKlf15* throughout adulthood. In mammals, *Klf15* expression is positively regulated by retinoic acid [13] and glucocorticoids [33]. Retinoid signalling affects eye development and tissue regeneration during larval development in *Drosophila* [34, 35]. Insects do not synthesise glucocorticoids, however analogous endocrine systems do exists in the form of the steroid hormone ecdysone, a hormone critical to many aspects of insect development. It is therefore plausible that *dKlf15* gene expression is modulated by endocrine signalling pathways similar to those in mammals. It will be important to elaborate the pathway(s) controlling *dKlf15* expression in order to identify both upstream regulators of *Klf15* expression and the gene’s downstream effectors as these may provide insights into human KLF15 function.

It is well established that mutations affecting nephrocyte function reduce survivorship of *Drosophila* larvae that ingest silver nitrate [5, 21]. Given that pericardial nephrocyte attrition occurred during larval development in *dKlf15* ^*NN*^ mutants, the observed sensitivity to silver nitrate was expected. In contrast, survivorship of adult mutants given silver nitrate was not different to that of controls fed the same diet, despite both genotypes showing sensitivity to the xenotoxin. This argues that nephrocytes are the primary system for mitigating xenotoxin insults in larvae but that other cells compensate for the loss in adults. It has been reported that the ablation of post-embryonic pericardial nephrocytes shortens adult lifespan [21], but this was not recapitulated in the present study. Because lifespan is influenced by genetic background [36], genetic differences in the present studies were minimised by extensive backcrossing. Whether this was also done in the post-embryonic nephrocyte ablation model is unclear but it may explain the discrepancy between the studies.

Pericardial nephrocytes are thought to modulate cardiac function [37], and preliminary studies suggest heart function is disrupted by *dKlf15* loss of function. This is an important area to follow-up using this model because mechanisms affecting cardiac function in *Drosophila* can be of direct relevance to human cardiovascular physiology [38]. It is also known that nephrocytes modulate the immune response by clearing circulating Necrotic protein [33]. Beyond this observation, it is not known to what extent insect nephrocytes control circulating endogenous macromolecules. Given the intractability of vertebrate models with which to study the clearance and turnover of circulating proteins by scavenger cells within the reticuloendothelial system, the fly and genetic ablation of nephrocyte using *dKlf15*-loss of function, offers an attractive alternative for such studies.

In summary *dKlf15*, is defined as a nephrocyte-restricted transcription factor critically required for the differentiation and sustained function of insect nephrocytes. As nephrocytes are important model of human renal podocytes the model has great potential to guide and support research into podocytopathies [39], an important class of kidney pathologies relevant to human cardiovascular health.

## Materials and Methods

### Strains used in this study

The *Canton Special* (*CaS*), *w* ^*1118*^ (used as wild-type strains in this study), *Bteb2* ^*f06447*^ (CG2932, FBgn0025679; called *dKlf15* ^*NN*^ in this study), *Dorothy-Gal4* (*Dot-Gal4*, originally described in [19], *UAS-mCherry* (TRiP control line), *Tub-Gal80* ^*ts*^, *Dp(1;3)122*, *Dp(1;3)120*, *Dp(1;3)473* and *Df(1)ED6727* lines were all from the Bloomington Stock Centre (Bloomington, USA, IL). The *HandC-Gal4* (*Hand-Gal4*) line was described in [20]). The *Hand-GFP* line was from Dr. Zhe Han (described in [24]). The two RNAi lines for knocking-down *Bteb2* / *dKlf15* were from the Vienna Drosophila RNAi stock Centre (with a targeting hairpin inserted into the second chromosome, VDRC) and Bloomington (*Bteb2* ^*JF02420*^, a Harvard TRiP line with a *Bteb2* targeting hairpin inserted into the third chromosome). All genetic combinations were generated by standard crosses. The *sns-mCherryNLS* line was kindly provided by Susan Abmayr and is described in [40]. Generation of TARGET flies (*Hand-Gal4*; *Tub-Gal80* ^*ts*^) was achieved by standard crossing techniques [26].

### Husbandry, propagation of flies, survivorship on silver nitrate and determination of lifespan

Flies were propagated routinely on a standard cornmeal-yeast diet at 25°C under a 12hr:12hr light-dark schedule. For TARGET experiments flies were reared at 18°C and then transferred to 29°C within 1–5 days of eclosing. Flies remained at 29°C for one to two weeks. Prior to analysis of heart function flies were transferred to 25°C for 24 hours. To reduce the effect of genetic background the *dKlf15* ^*NN*^ mutant was backcrossed onto the *w* ^*1118*^ for >20 generations. Fur establishing survivorship of larvae on silver nitrate, 20 eggs from control or *dKlf15* ^*NN*^ mutants were placed onto agar with yeast paste containing different concentrations of silver nitrate. Survivorship was calculated as the percentage of adults eclosing. For the determination of lifespan, male or female flies were collected within 1–3 days of eclosion and housed in vials with free access to food (cornmeal-molasses diet) in groups of 15. Flies were tipped to fresh food twice a week and the number of dead flies recorded until all flies were dead.

### Generation of antibodies to dKlf15 and dAmnionless

Antisera were prepared by GenoSphere Biotechnologies (Paris, France). The immunogen chosen for dKlf15 was the peptide CPPDLSDWEQRLLDN, whereas the peptide for dAmnionless was DPRLWRWRHLGLRLR. Antisera to the peptides were generated in rabbits (dKlf15) and guinea pigs (dAmnionless). Antisera were affinity purified to yield the total IgG fraction. Antibodies were verified for specificity by ELISA using the peptide as target antigen. Pre-incubating antibodies with a 10-fold excess of peptide blocked all immunoreactivity in adult flies. Anti-dKlf15 antisera was used at 1:10 to detect protein in wild-type flies and 1:100 in flies over-expressing *dKlf15*. Anti-Amnionless antisera were used at 1:100.

### Generation of transgenic *dKlf15-Gal4* flies

To generate the *dKlf15-Gal4* line, a 2131bp fragment of DNA containing sequence upstream of the predicted 5’ translation initiation site of the *dKlf15* gene was cloned into pPTGAL4 and transgenic flies generated by a commercial company (BestGene Inc, CA, USA).

### rtPCR

PCR was performed as described previously [41]. Primers for detecting *dKlf15* gene expression were as follows: (left) GGAGGAGAGCAACAGCAATC; (right) CATCGTGTCCTTGGAATGTG. This amplified a 538bp amplicon, between base pairs 420 and 957 of the 962bp *dKlf15* transcript. In the *dKlf15* ^*NN*^ mutant this region is downstream of the P-element insertion (inserted at the 311^st^ bp; see red bars in Fig 2A). The *dKlf15* gene has no introns, so genomic DNA was reduced by DNase treatment. No amplicons were detected in cDNA prepared from whole wild-type adult flies, ovaries or larvae, so isolated hearts were used to prepare cDNA to establish expression in adult flies.

### Imaging the larval and adult heart

Larvae were anaesthetised with Flynap (Carolina Biological Supply Company, Burlington, NC, USA) for 30 minutes, placed dorsal side up, on a microscope slide and then cover-slipped so as to partially flatten but not burst the larvae. Adult were dissected and hearts stained as described previously [41]. For some experiments, vital dyes were used to identify functional nephrocytes or test their endocytic function (wheat germ agglutinin at 1 μg/mL for 15 minutes or 50 μg/mL 10kDa fluorescently labelled dextran for 0–30 minutes). Semi-intact preparations were then washed three times, fixed for 20 minutes with 1% formaldehyde and co-stained with antibodies (and then the relevant secondary antibodies) or Hoechst to visualise DNA and then imaged.

### Immunostaining in embryos

Eggs were collected from agar plates every 24h. Embryos were dechorionized in 50% bleach and fixed as described before [42]. Primary antibodies used were: rabbit anti-Tin (1:800) [43], rabbit anti-Odd (1:200) [44], mouse anti-Eve (1:5)(Developmental Studies Hybridoma Bank) and guinea pig anti-β3-Tubulin (1:1.000)[45]. Confocal stacks were acquired using a Zeiss LSM 5 Pascal and pictures were processed using ImageJ. Cells were counted in confocal stacks of stained embryos of both genotypes at stage 17. To test the statistical significance of the acquired numbers an unpaired, two-tailed student’s t-Test was used.

### Tissue preparation and TEM imaging

Pericardial nephrocytes in third instar larvae were analysed by transmission electron microscopy (TEM) in semi-dissected animals. Therefore, larvae were collected, dissected in cold PBS and fixed in 3.7% formaldehyde for 1h at room temperature. Further fixation and preparation for TEM imaging was essentially done as described in [46]. Samples were analysed using a 60kV Zeiss 902 electron microscope and images were processed using ImageJ and Photoshop CS.

### Analysis of endocytosis using 10kDa dextran^488^

Adult flies were anaesthetised by brief exposure to CO_2_ and then dissected as for heart analysis (see above). The semi-intact preparation was then incubated in a solution of Alexa^488^ fluorescently labelled 10kDa dextran (made with Hanks basic saline solution (HBSS, Sigma, Dorset UK) containing 2mM CaCl_2_), at 25°C for 0–30 minutes. Endocytosis was then inhibited by placing the preparation on ice, washing with ice-cold HBSS and fixing samples for 15 minutes with 4% formaldehyde in HBSS. Some samples were co-incubated with wheat-germ agglutinin (WGA) conjugated to the Alexa^594^ fluorochrome.

### Epifluorescence and confocal microscopy and image analysis

Fluoresce microscopy of flies was performed with a Zeiss Axioskop MOT II microscope (Carl Zeiss, Welwyn Garden City, UK) illuminated with mercury or halogen light sources for fluorescence or differential interference contrast (DIC) imaging using a 40x objective. Images were captured with an ORCA-ER CCD camera (Hamamatsu Photonics KK, Japan; Welwyn Garden City, UK) coupled to Openlab 4.1 (Improvision, Coventry, UK). Confocal images were collected with a Zeiss LSM 780 coupled to Zen imaging software. Images were coloured, contrast enhanced and overlaid using Photoshop CS3. All micrographs from an experiment were collected using the same microscope settings and image alterations. For quantification of fluorescence, micrographs were opened in ImageJ and converted to 8-bit greyscale format. The line tool was then used to draw across a region of interest and the maximum pixel intensity recorded.

### Statistics

When more than two genotypes or treatments were used in an experiment one-way ANOVA was used to test the hypothesis that genotype may have affected heart function and post hoc test (Tukey’s HSD) was used to establish *P* values between control and the different genotypes. If only two genotypes or treatments were used an unpaired two-tailed student’s t-Test was applied to establish the *P*-value. For endocytosis experiments a 2-way ANOVA was used to examine if there was an interaction between the fluorescence signal, time and genotype.

## Supporting Information

S1 Fig Electronic pipeline data making case for renaming Bteb2 to dKlf15. (DOCX)

S2 Fig High throughput data showing *dKlf15* expression in embryo and adult. (DOCX)

S3 Fig Immunoreactivity of anti-dKlf15 antisera is restricted to pericardial nephrocytes. (DOCX)

S4 Fig *dKlf15-Gal4* driven *UAS-RedStinger*. (DOCX)

S5 Fig Anti-dKlf15 antisera detect dKlf15 in wild type but not *dKlf15* ^*NN*^ L3 larvae. (DOCX)

S6 Fig Sns and Hand reporter expression in L2 larvae. (DOCX)

S7 Fig Localisation of fluorescently labelled 10 kDa dextran in wild type and *dKlf15* conditionally-silenced adult nephrocytes. (DOCX)
