# Supplementary material for: Klf15 Is Critical for the Development and Differentiation of Drosophila Nephrocytes
Source: PLoS One. 2015 Aug 24;10(8):e0134620. doi: 10.1371/journal.pone.0134620 (PMC4547745; doi:10.1371/journal.pone.0134620)

**S1 Figure. Electronic pipeline data making case for renaming Bteb2 to dKlf15.**

(A) Genetree showing prediction that *Drosophila Bteb2* gene is more closely linked to human *KLF15* than mammalian *Bteb2*. (B) Schematic showing domain structure of *Bteb2 / dKlf15*. (C) Amino acid sequence similarity is greater between *Drosophila* dKlf15/ Bteb2 and human KLF15 than it is between the fly gene and human BTEB2. (D) Alignment of DNA binding domains showing the specific sites conserved between *Drosophila* dKlf15 / Bteb2 and human KLF15.


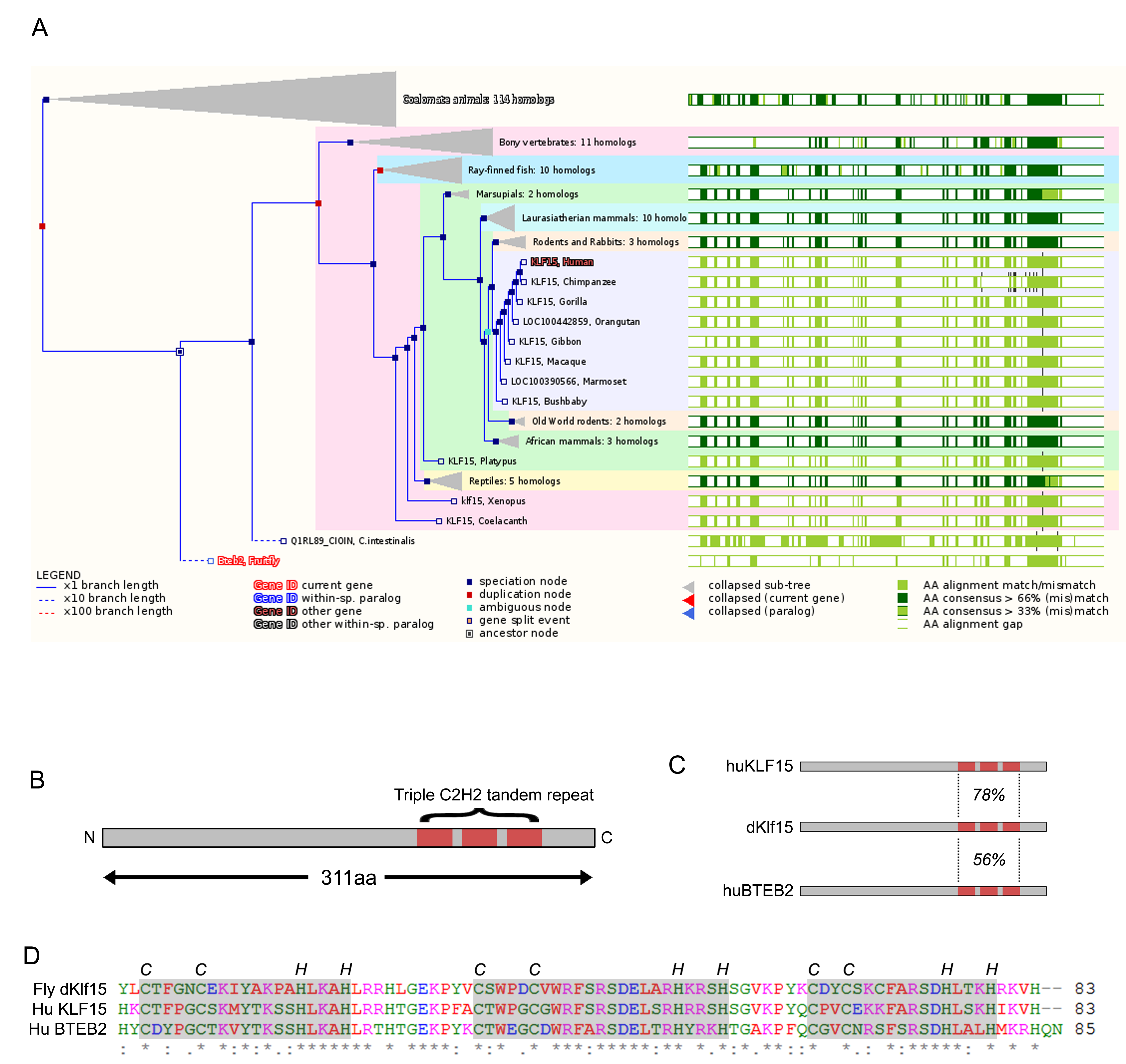

Supplement: S1 Fig — (DOCX) [file pone.0134620.s001.docx]
