# Supplementary material for: Klf15 Is Critical for the Development and Differentiation of Drosophila Nephrocytes
Source: PLoS One. 2015 Aug 24;10(8):e0134620. doi: 10.1371/journal.pone.0134620 (PMC4547745; doi:10.1371/journal.pone.0134620)

**S2 Figure. High throughput data showing *dKlf15* expression in embryo and adult**.

(A) *In situ* data indicate presence of maternal *dKlf15* transcripts in embryos before stage 7-8 and mRNA expressed by embryonic garland cells after stage 10. (B) FlyAtlas data showing moderate expression of *dKlf15* in the adult heart. The signal in the heart was recorded in 4/4 arrays, other expression signals were not reliably recorded in multiple arrays.


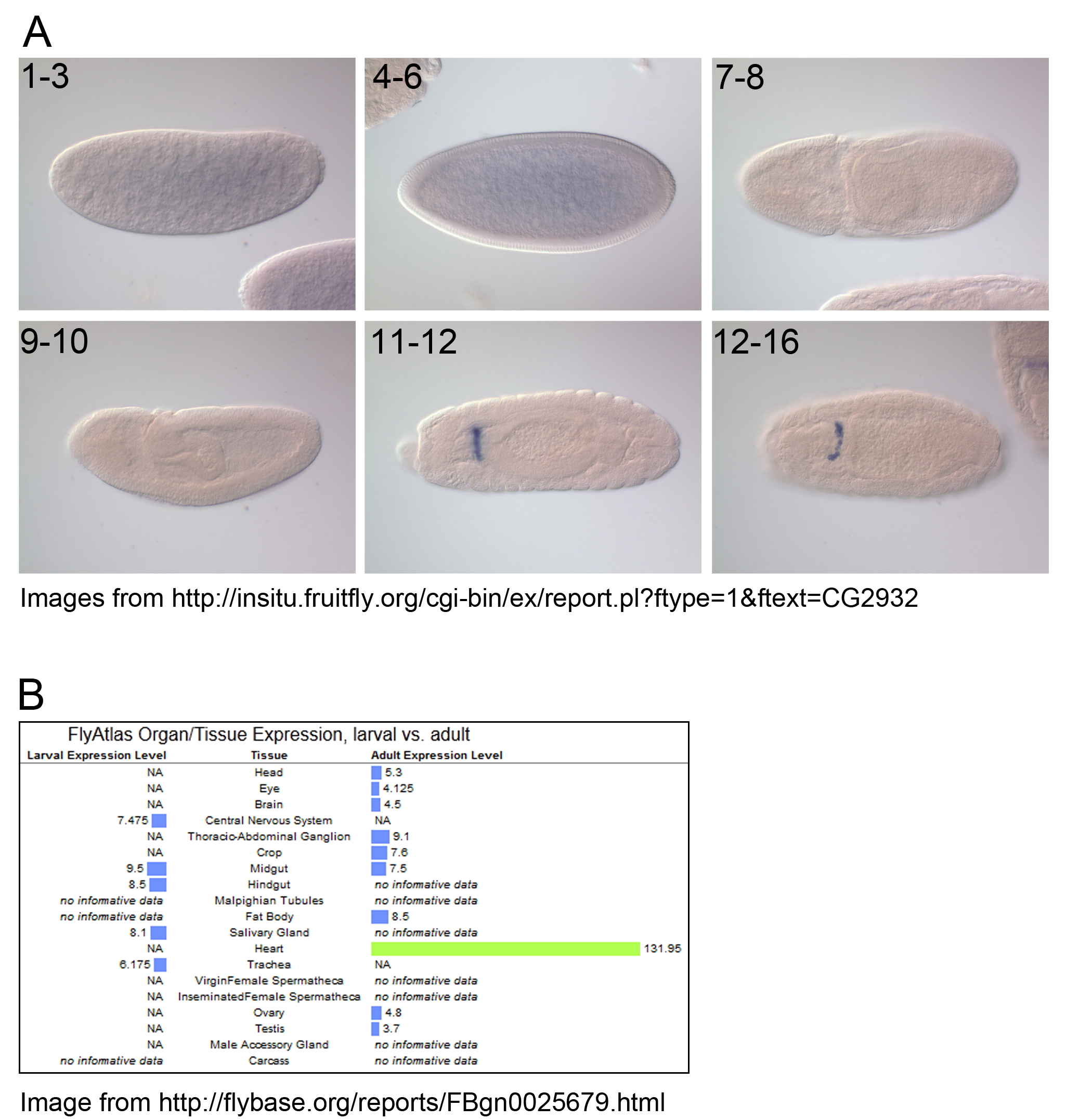

Supplement: S2 Fig — (DOCX) [file pone.0134620.s002.docx]
