# Supplementary material for: Klf15 Is Critical for the Development and Differentiation of Drosophila Nephrocytes
Source: PLoS One. 2015 Aug 24;10(8):e0134620. doi: 10.1371/journal.pone.0134620 (PMC4547745; doi:10.1371/journal.pone.0134620)

**S3 Figure. Immunoreactivity of anti-dKlf15 antisera is restricted to pericardial nephrocytes**.

Adult tissues were stained with wheat germ agglutinin (WGA; red), Hoechst (blue) and anti-dKlf15 antisera (1:10) and imaged by confocal microscopy. Immunoreactivity was observed in the nuclei of pericardial nephrocytes (PNs) but not the nuclei of adult fat body cells, the ovary or oenocytes.


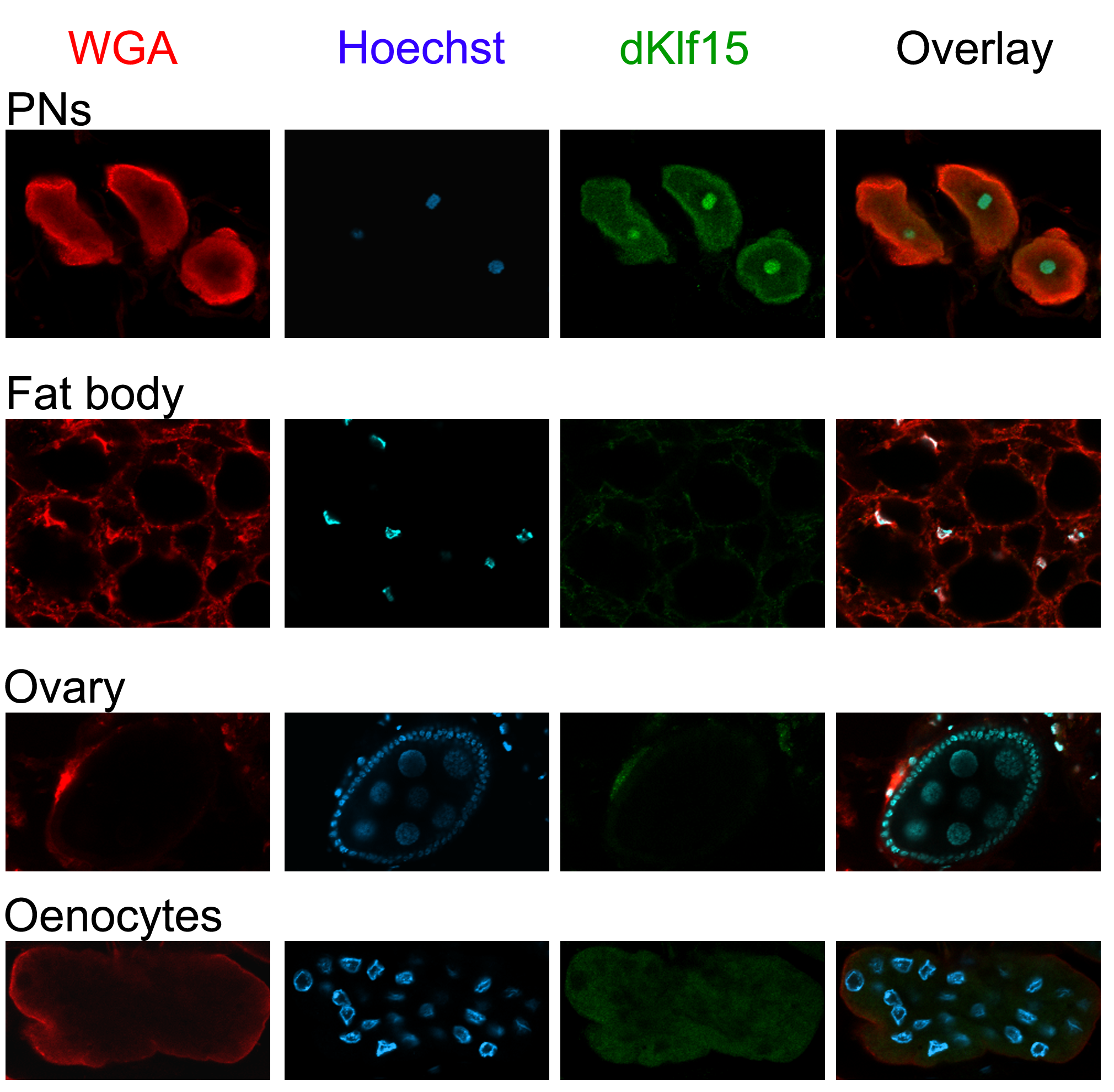

Supplement: S3 Fig — (DOCX) [file pone.0134620.s003.docx]
