# Supplementary material for: Klf15 Is Critical for the Development and Differentiation of Drosophila Nephrocytes
Source: PLoS One. 2015 Aug 24;10(8):e0134620. doi: 10.1371/journal.pone.0134620 (PMC4547745; doi:10.1371/journal.pone.0134620)

**S4 Figure. *dKlf15-Gal4* driven *UAS-RedStinger***.

Expression of RedStinger driven by 2131bp of the putative *dKlf15* enhancer region. No expression was observed in L1 larvae, whereas expression was localised to pericardial cells in the L2 larvae.


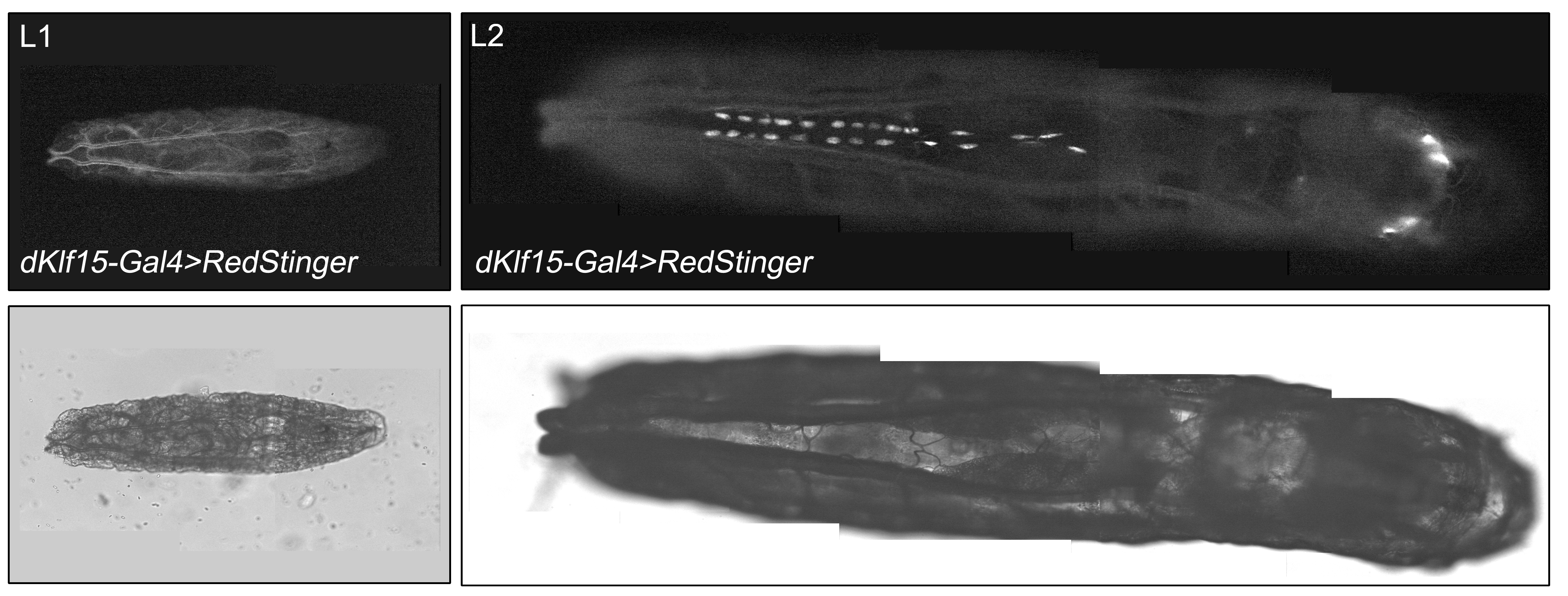

Supplement: S4 Fig — (DOCX) [file pone.0134620.s004.docx]
