# Supplementary material for: Klf15 Is Critical for the Development and Differentiation of Drosophila Nephrocytes
Source: PLoS One. 2015 Aug 24;10(8):e0134620. doi: 10.1371/journal.pone.0134620 (PMC4547745; doi:10.1371/journal.pone.0134620)

**S5 Figure. Anti-dKlf15 antibodies detect dKlf15 in wild type but not *dKlf15^NN^* L3 larvae**.

Upper panels show wild type (WT) pericardial nephrocytes (arrows) stained with phalloidin (red) to identify the heart and anti-dKlf15 antisera (green) which localises to the pericardial nephrocytes’ nucleus. In contrast, no anti-dKlf15 staining can be seen in the degenerating pericardial nephrocytes in *dKlf15^NN^* mutants (multinucleate cells, arrowheads in lower panels). HT = Heart tube; Tr = Trachae.


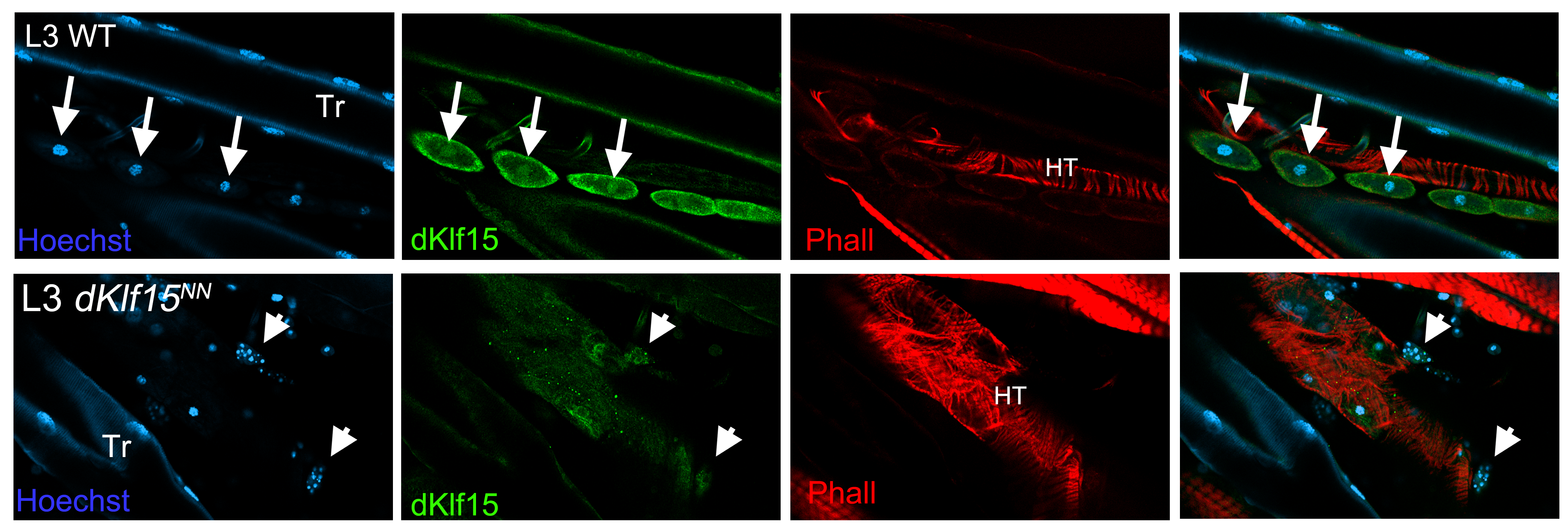

Supplement: S5 Fig — (DOCX) [file pone.0134620.s005.docx]
