# Supplementary material for: Klf15 Is Critical for the Development and Differentiation of Drosophila Nephrocytes
Source: PLoS One. 2015 Aug 24;10(8):e0134620. doi: 10.1371/journal.pone.0134620 (PMC4547745; doi:10.1371/journal.pone.0134620)

**S6 Figure. *Sns* and *Hand* reporter expression in L2 larvae**.

*Hand-GFP* wild type (*dKlf15^+/+^*) and *dKlf15^NN^* lines expressing a *sticks and stones* reporter (red). Images show a region of the distal larval heart in L2 larvae. *Sns* gene expression reporter can be seen in the pericardial nephrocytes of wild type (one is depicted by the arrow) but not in the mutants’ nephrocytes (the arrowhead in the lower panel indicates a mutant pericardial nephrocyte adjacent to Hand-GFP positive cardiomyocytes). In contrast, expression of the Sns reporter is seen in non-heart cells in both genotypes (asterisks).


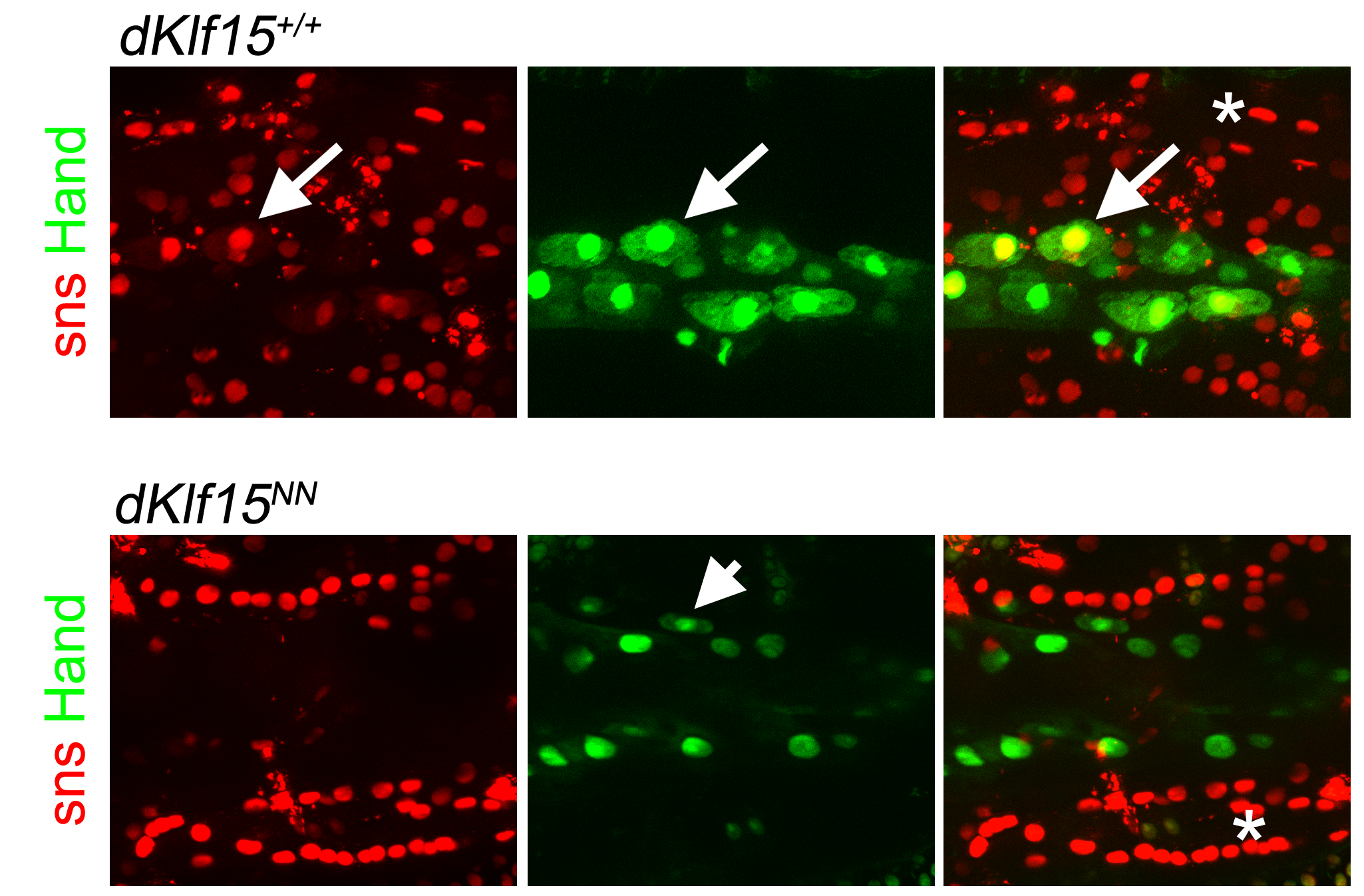

Supplement: S6 Fig — (DOCX) [file pone.0134620.s006.docx]
