# Supplementary material for: Klf15 Is Critical for the Development and Differentiation of Drosophila Nephrocytes
Source: PLoS One. 2015 Aug 24;10(8):e0134620. doi: 10.1371/journal.pone.0134620 (PMC4547745; doi:10.1371/journal.pone.0134620)

**S7 Figure. Localisation of fluorescently labelled 10 kDa dextran in wild type and *dKlf15* conditionally-silenced adult nephrocytes.**

*dKlf15* was silenced in adult nephrocytes using the TARGET system as described in the methods. Nephrocytes in which *dKlf15* had been silenced were unable to bind dextran whereas controls and flies in which *dKlf15* had not been silenced were able to accumulate dextran.


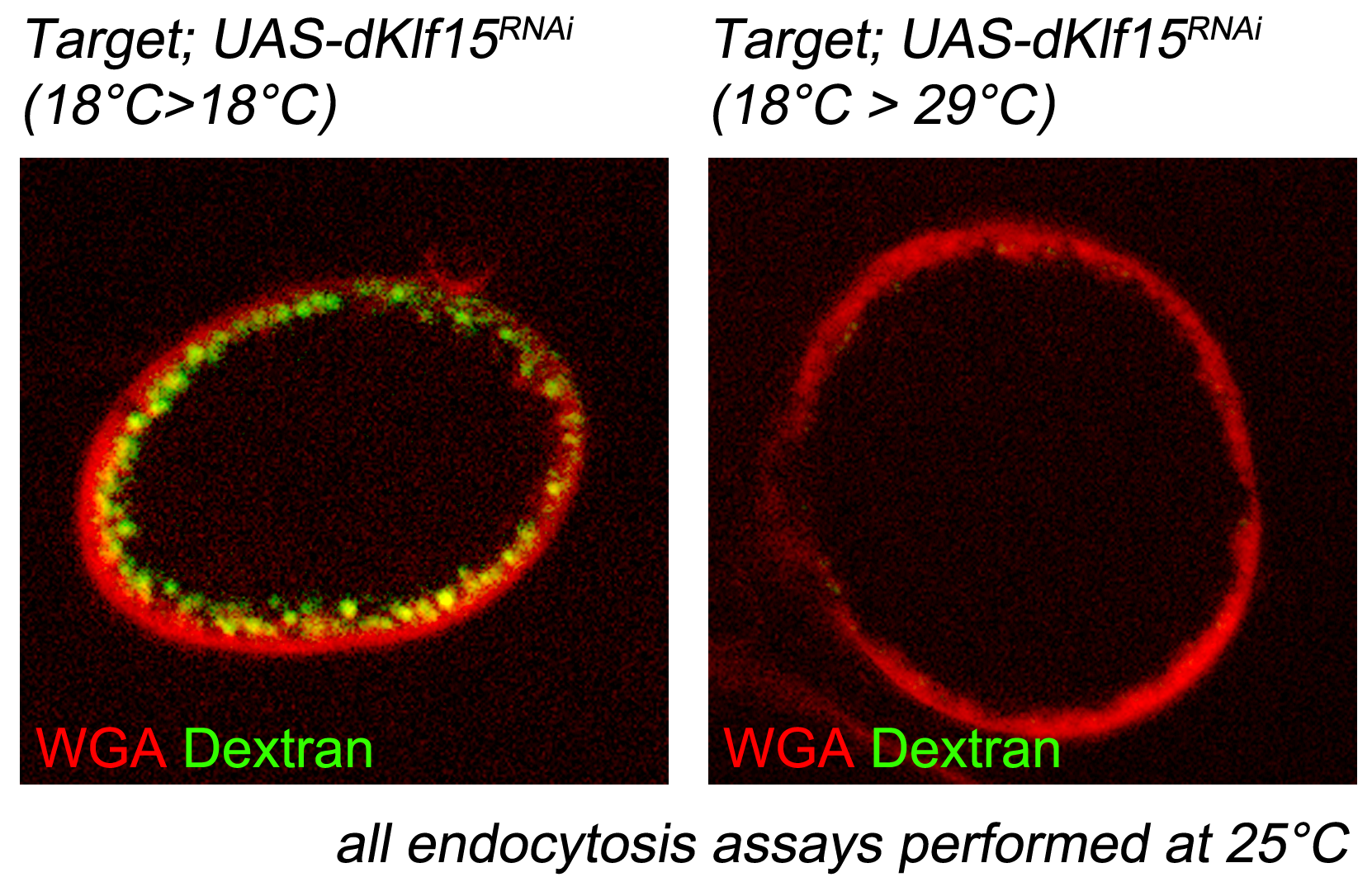

Supplement: S7 Fig — (DOCX) [file pone.0134620.s007.docx]
